# Special Issue “Antimicrobial Resistance: From the Environment to Human Health”

**DOI:** 10.3390/microorganisms9040686

**Published:** 2021-03-26

**Authors:** Carla Viegas, Susana Viegas

**Affiliations:** 1 H&TRC-Health & Technology Research Center, ESTeSL-Escola Superior de Tecnologia da Saúde, Instituto Politécnico de Lisboa, 1990-096 Lisbon, Portugal; suana.viegas@ensp.unl.pt; 2 NOVA National School of Public Health, Public Health Research Centre, Universidade NOVA de Lisboa, 1990-096 Lisbon, Portugal; 3 Comprehensive Health Research Center (CHRC), 1169-056 Lisbon, Portugal

Since the 1940s, humans have developed new drugs and consumption has increased significantly in the last 15 years. This global phenomenon is now a major public health concern due to Antimicrobial resistance (AMR). AMR is a severe threat to both human and animal health. Indeed, the spread across systems can occur through a number of pathways, both related and unrelated to agriculture, comprising the wastewater, soils, manure applications, direct exchange between humans and animals, and food consumption. Characterizing the presence in the environment (baseline assessment of the AMR prevalence) and managing human health risks due to exposure to resistant organisms requires national and international interdisciplinary cooperation. In fact, a holistic approach such as the One Health approach is needed to tackle this public health menace. This Special Issue includes 19 papers (16 articles and 3 reviews) that collectively provide novel information about this topic.

In one of the published studies, a method was developed to detect bacteria resistant to colistin, carbapenems, and β-lactams in commercial poultry farms, characterizing also the phylogenetic and virulence markers of *E. coli* isolates to assess virulence risk, and to evaluate potential transfer of AMR. The study allowed concluding about the presence of multidrug resistance to the last-resort antibiotics that are transferable between bacteria in food-producing animals [1]. Also, in a different animal production environment, 145 florfenicol-resistant enterococci, isolated from swine fecal samples collected from 76 pig farms, were investigated for the presence of optrA, cfr, and poxtA genes by PCR. In this study it was possible to observe a dissemination of linezolid resistance genes in enterococci of swine origin in Central Italy and confirm the spread of linezolid resistance in animal settings [2]. In another study, the pathogenomics of carbapenem-resistant *Aeromonas veronii* (*A. veronii*) isolates recovered from pigs in KwaZulu-Natal, South Africa were explored by whole genome sequencing on the Illumina MiSeq platform. In this case, phylogenomic and metadata analyses revealed a predilection for water environments and aquatic animals, with more recent reports in humans and food animals across geographies, making *A. veronii* a potential One Health indicator bacterium [3]. An extensive culturomics approach was applied in 27 healthy pigs from seven different farms, in a different study, and a high frequency of methicillin-resistant staphylococci supporting the need for enhanced efforts within the “One Health” approach to prevent and manage the antibiotic resistance crisis in the human and veterinary medicine sectors was observed [4].

Another study also published in this special issue addresses how soil disturbance, and the subsequent shift in community composition, will affect the types, abundance, and mobility of antibiotic resistance genes (ARGs) that compose the active layer resistome by assessing resistance phenotypes through antibiotic susceptibility testing, and analyzing types, abundance, and mobility of ARGs through whole genome analyses of bacteria isolated from a disturbance-induced thaw gradient in Interior Alaska. This study emphasizes the hypothesis that both phylogeny and ecology shape the resistome and suggest that a shift in community composition as a result of disturbance induced will be reflected in the predominant ARGs comprising the active layer resistome [5]. In a different study, several antibiotic-resistant bacteria (ARB) were isolated from the mangroves in Kerala, India showing that MDR with strong biofilm formation is prevalent in natural habitat and if acquired by deadly pathogens may create a highly negative impact in public health [6].

Using water as an environmental matrix, one study aimed at characterizing and tracking nonresistant and extended-spectrum β-lactamase–producing (ESBL)-*Salmonella* spp. from agricultural settings to nearby water sources, highlighting their antibiotic resistance genes (ARG) and virulence factor (VF) distribution using a combination of both culture-dependent and independent methods, concluding that agricultural environment contamination may have a direct relationship with the presence of antibiotic-producing *Salmonella* in freshwater streams [7]. Also, in a different study was investigated the persistence of an (ESBL)-producing *Escherichia coli* (*E. coli*) pEK499 and its clinically most important ARG (bla_CTX-M-15_), after introduction via irrigation water or manure into a lettuce-growing system. This study demonstrated long-term persistence of undesired ARB and ARG after their introduction via both irrigation and amendments adding the need to define critical actions in order to mitigate their transfer to the consumer [8]. Irrigation water was also used to screen ESBL-producing *E. coli, Enterobacter cloacae*, and *Citrobacter freundii* for their potential to transmit resistance to antibiotic-susceptible *E. coli,* proving that ESBL-producing *E. coli* was able to transfer resistance with different efficiency to *E. coli*-susceptible recipients [9].

One of the published studies aimed to compare antimicrobial resistance (AMR) in extended-spectrum cephalosporin-resistant and generic *E. coli* from a One Health approach applied on a beef production system, identifying the municipal sewage as a hot spot for MDR emergence and dissemination [10]. Also published was a study that identified associations in resistance traits between *E. coli* isolated from clinical, dairy manure, and freshwater ecosystem environments concluding that manure and environmental isolates were significantly different from clinical isolates based on analyzed traits, suggesting more transmission occurs between these two sources in the sampled environment [11]. The same approach was held by a different study aiming at detecting several virulence factors genes (fimA, papC, papG III, cnf1, hlyA and aer) and to determine the conjugative capacity in a wide collection of ESBL-producing *E. coli* isolated from different sources (human, food, farms, rivers, and wastewater treatment plants), emphasizing the need of a specific surveillance program of AMR indicators in wastewaters from animal or human origin, in order to apply sanitary measures to reduce the burden of resistant bacteria arriving to risky environments such as WWTPs [12].

An additional study published intended to assess settleable dust loading rates and bioburden in Portuguese dwellings by passive sampling onto quartz fiber filters and electrostatic dust cloths (EDCs), including azole-resistance screening reinforcing the importance of applying azole resistance screening to unveil azole resistance detection in fungal species [13]. A similar approach was applied in a different study developed in two small commercial bakeries and in a pizzeria, which aimed to measure occupational exposure to flour and microorganisms, including azole resistance screening where the presence of azole-resistant fungi in these specific occupational environments was detected [14].

Another published study observed three major subtypes (FimH41, H22, and H30) of ST131 in fecal carriage in dogs in Taiwan by rectal swabs used for *E. coli* isolation from non-infectious dogs in a veterinary teaching hospital in Taiwan, finding three major subtypes (FimH41, H22, and H30) of ST131 in fecal carriage [15].

A multicenter retrospective study applied in two different teaching hospitals in Romania was published. This study analyzed urine samples from patients to determine the frequency of incriminating pathogens and their resistance to different antibiotics, concluding that the obtained data are an important tool in managing urinary tract infections [16].

The three review papers also published in this special issue focused on three different topics: Mitochondria-mediated azole drug resistance and fungal pathogenicity addressing the opportunities for therapeutic development [17]; correlation between exogenous compounds and the horizontal transfer of plasmid-borne antibiotic resistance genes [18]; and, also Antibiotic resistance profiles, molecular mechanisms, and innovative treatment strategies of *Acinetobacter* [19], all presenting crucial updates of each topic and the current progress concerning innovative strategies for combating multidrug-resistant species.

Overall, the papers in this Special Issue reveal different perspectives of antimicrobial resistance: from the presence of resistant organisms in different environmental compartments to human exposure in different settings. Furthermore, descriptions of prevention and intervention actions to tackle this public health menace were also described and can be used in the future.
